# Coping motives mediate the relationship between PTSD and MDMA use in adolescents with substance use disorders

**DOI:** 10.1186/s13722-022-00329-y

**Published:** 2022-09-04

**Authors:** Lukas Andreas Basedow, Melina Felicitas Wiedmann, Veit Roessner, Yulia Golub, Sören Kuitunen-Paul

**Affiliations:** 1 Department of Child and Adolescent Psychiatry, Faculty of Medicine, Technische Universität Dresden, Dresden, Germany; 2 Division of Clinical Psychology and Psychotherapy, Dept. of Psychology, Philipps-University of Marburg, Marburg, Germany; 3 Chair for Clinical Psychology and Psychotherapy, Technische Universität Chemnitz, Chemnitz, Germany

**Keywords:** Addiction, Drugs, Ecstasy, Self-medication, Trauma disorders

## Abstract

**Background:**

Post-traumatic stress disorder (PTSD) and substance use disorders (SUDs) often co-occur in adolescent patients. Previous research has shown that these patients differ from SUD patients without PTSD in terms of their substance use patterns. In this study, we aimed to test whether substance use in this population is related to an attempt to self-medicate PTSD-related symptoms.

**Methods:**

German adolescent patients (aged 13–18 years) at an outpatient clinic for SUD treatment, *n* = 111 (43% female), completed a self-designed questionnaire on use motives, a measure of PTSD-related experiences, and underwent a standardized psychiatric interview including structured substance use questions. Participants were subsequently classified as ‘no traumatic experiences (‘noTEs’ but SUD), ‘traumatic experiences but no current PTSD diagnosis’ (‘TEs’ with SUD), and ‘PTSD’ with SUD. After establishing a self-designed motive measurement through exploratory and confirmatory factor analyses, we calculated non-parametric group differences and a mediation analysis in a linear regression framework.

**Results:**

The past-year frequency of MDMA use was highest in the PTSD group and lowest in the noTE group (*H* (2) = 7.2, *p* = .027, *η*^*2*^ = .058), but no differences were found for frequencies of tobacco, alcohol, cannabis, or stimulant use (all *H* ≤ 4.9, *p* ≥ .085, η^*2*^ ≤ .033). While controlling for sex, the three groups showed a similar pattern (highest in the PTSD group and lowest in the noTE group) for coping scores (*F* (103) = 5.77, *p* = .004, *η*^*2*^ = .101). Finally, mediation analyses revealed an indirect effect of coping score (*b* = 0.61, 95% CI [0.29, 1.58], *p* = .145) on the association between group membership and MDMA use frequency.

**Conclusions:**

In adolescent SUD patients, we found an association of current PTSD and lifetime traumatic experiences with higher MDMA use that could be partially explained by substance use being motivated by an attempt to cope with mental health symptoms. This indicates a coping process involved specifically in MDMA use compared to the use of other psychoactive substances, possibly due to unique psychoactive effects of MDMA.

**Supplementary Information:**

The online version contains supplementary material available at 10.1186/s13722-022-00329-y.

## Background

Post-traumatic stress disorder (PTSD) has been linked repeatedly to chronic substance use as well as substance use disorders (SUDs), such that psychiatric patients with either disorder often fulfil diagnostic criteria for the other one as well [1–5]. For example, 20–54% of adolescent SUD patients fulfil PTSD criteria [6, 7], while 30% of adolescent PTSD patients present with SUD [8]. Similar patterns have been shown for adult patients [2, 5] as well as adolescents [1, 3, 4]. Several explanations have been hypothesized for these findings. First, a common biological dysfunction or vulnerability might increase the likelihood to develop either disorder in the course of their life [9] (given the appropriate environmental variables, such as a traumatic events), as indicated by data showing that both disorders have a similar age of onset, namely around adolescence [10]. Accordingly, some studies have found genetic markers that are related to PTSD as well as SUD, such as polymorphism of the GABA receptor [11–13]. Second, circumstances promoting development of SUD in adolescents often include adverse life events or traumatic experiences (TEs) [14], and this increased exposure to TEs might also facilitate the development of PTSD [15, 16]. Finally, a co-occurrence of PTSD and SUD might be a result of substance use as a coping mechanism for dealing with PTSD symptoms [17]. Dealing with PTSD symptoms like hyperarousal, avoidance, or intrusion [18] presents a challenge for adolescents that is happening during the same time period in life where experimentation with psychoactive substances is most likely [19]. It is likely, that some substances can alter the acute experience of the PTSD symptoms in a sense that promotes future substance use, i.e., through negative reinforcement [20, 21], which increases the risk of experimental or recreational use developing into a problematic pattern as exhibited by the presence of a SUD. The hypothesis suggesting this pattern of development is commonly referred to as the self-medication hypothesis [22, 23].

The latter has been repeatedly invoked in the description of the PTSD-SUD relationship [24–27]. While previous research has focussed on adult patients with PTSD and SUD who reported coping motives with regard to their substance use [17], or on adolescents without SUD or PTSD [28] little research has been conducted to directly explore the relationship between PTSD, substance use, and coping motives. In two studies with adult participants drawn from the general population, it has been shown that coping motives act as mediator in the relationship between TEs and problematic substance use [29, 30]. In contrast, one study in adolescent SUD patients has shown that coping motives are increased in participants with co-occurring SUD and PTSD, instead of TEs alone [31].

In the present study, we aim to explore the relationship between adolescent PTSD and SUD in the context of the self-medication hypothesis. To do so we investigate differences in reported coping motives between adolescents with a SUD, adolescents with a SUD and TEs but no PTSD, and adolescents with a SUD and PTSD. Additionally we explore differences in past-year substance use between these three groups and aim to understand the connection between these three variables (PTSD group, coping motives, and substance use frequency) through a mediation analysis. Given the substance-specific psychoactive effects that might interact with PTSD symptoms, we conduct analyses for several substances, but restrict the mediation analyses to those substances whose use differed between groups.

## Methods

### Participants

Between November 2017 and April 2021, *n* = 303 treatment-seeking adolescents at a German outpatient clinic for adolescents with SUD consented to participate in the study. Since our sample consists of adolescent patients, the main driver for treatment-seeking are the wishes of their parents. This setting of borderline involuntary treatment leads to very low motivation on the side of the adolescents to participate in additional effort regarding research participation. Therefore only *n* = 162 (41% female) participants filled out the use motives questionnaire and were selected for exploratory and confirmatory factor analyses. For the main analysis, participants were selected who had answered at least 80% of the items in the relevant questionnaires (*n* = 111, 43% female). Participants were divided into three groups according to their trauma status resulting from the PTSD questionnaire: no history of traumatic experiences (‘noTEs’), a history of traumatic experiences but no PTSD (‘TEs’), and past-year PTSD (‘PTSD’).

### Materials

#### TEs and PTSD

The University of California at Los Angeles Post Traumatic Stress Disorder Reaction Index for DSM-IV [32], German version by [33], is a self-report questionnaire assessing TEs and PTSD symptoms in adolescents. The instrument contains a Criterion A section, in which patients select the lifetime TE that currently afflicts them the most. The next section assesses the frequency of occurrence of PTSD symptoms during the past month (rated from 0 = none of the time to 4 = most of the time). The items map directly onto the DSM-IV intrusion (Criterion B), avoidance (Criterion C), and hyperarousal (Criterion D) symptom clusters. PTSD is considered to be present when all four criteria (Criterion A, B, C, & D) are fulfilled [32]. Outcomes for this questionnaire were presence of a PTSD (yes/no) and presence of a TE (yes/no).

#### Use motives

To assess use motives, we used a self-designed questionnaire asking twenty-two questions that are answered on a scale with zero (“never applies”), one (“rarely applies”), two (“sometimes applies”), three (“mostly applies”) or four (“always applies”) points. The questionnaire has been designed to provide details about a patients substance use and ten of the items allow for the extraction of three scores for different use motives: ‘coping’ (4 items), ‘social motives’ (3 items), and ‘other’ (3 items). A detailed overview over the ten use motive items of the questionnaire can be found in Additional file 1: Table S3. The remaining twelve items refer to the participants rating of their ability to control their drug use (e.g. “I have the feeling that I have no control over my drug use”; “I feel like I relapse often”), which are not analysed here. To determine if the theoretical structure of the questionnaire is empirically supported, we perform preliminary exploratory and confirmatory factor analyses. The main outcome is the combined score of the ‘coping’ items, with a maximum score of 16, and a higher score indicating more frequent substance use because of coping motives.

#### Comorbid diagnoses

The Mini-International Neuropsychiatric Interview for Children and Adolescents (MINI-KID) [34] is a structured diagnostic interview used to evaluate the presence of psychiatric disorders, according to DSM-5 criteria. All interviews were conducted by psychologists working in our department of adolescent substance abuse using a German translation of the original MINI-KID [35]. Outcomes were the presence of any SUD, psychotic, mood (major depression or bipolar disorder), anxiety (general anxiety disorder, panic disorder, agoraphobia, separation anxiety disorder, social phobia, specific phobia), behavioural (attention deficit hyperactivity disorder, conduct disorder, oppositional defiant disorder) or obsessive–compulsive (OCD) disorder. The MINI-KID was conducted in the context of a study registered at clinicaltrials.gov (NCT03444974), with all licensed administrations (invoice #20220315.1) conducted before March 15th 2022.

#### Substance use interview

The pattern of substance use was assessed via interview [36], asking for the number of days each substance was used per month over the past year. Outcome variables from this assessment were the presence (yes/no) of past-year use of tobacco, alcohol, cannabis, cocaine, benzodiazepines, opioids, solvents, methylenedioxymethamphetamine (MDMA), and stimulants (= amphetamine and methamphetamine), and the past-year use frequency of each substance in average number of use days per month. However, since none of our participants reported regular past-year use of cocaine, opioids, benzodiazepines or solvents we excluded these substances from the analyses.

### Procedure

Data collection was embedded into standard diagnostic procedures. During the first clinical appointment, participants as well as legal guardians were asked to provide written informed consent to the study. Questionnaires were handed out and substance use was evaluated by the hospital staff member (therapist, psychologist, or physician). The MINI-KID was conducted approx. 1–4 weeks later. The study was conducted in accordance with the Declaration of Helsinki and all procedures were approved by the Institutional Review Board of the University Hospital C. G. Carus Dresden (EK 66022018).

### Statistical analysis

#### Exploratory factor analysis

To account for the non-normal distribution of motive items (Shapiro–Wilk test for each questionnaire items *p* < 0.001), we used factor analysis extraction methods. The number of latent factors were explored with Scree plot, Kaiser-Guttman criterion, the revised MAP test as well as Parallel Analyses with principal components and raw data permutation [37]. Possible item-factor assignments (factor structures) were deducted with exploratory factor analyses (EFA) in IBM SPSS Statistics 27.0 using Principal Axis extraction with Promax-rotation (kappa = 4).

#### Confirmatory factor analysis

The adequacy of factor structures was tested for the given empirical data with confirmatory factor analysis (CFA) using the lavaan package [38] in RStudio [39]. We tested the theoretical model (the three factors, ‘coping’, ‘social motives’ and ‘other’ consisting of distinct items), the empirical model build upon the results from the EFA, and the combined model that integrates theoretical considerations into the empirical model using the diagonally weighted least-squares (DWLS) method of estimation to account for non-normality within the categorical items. A good absolute model fit would be indicated by a Χ^**2**^ to degrees of freedom ratio < than 2 (a ration between 2 and 3 is acceptable), a Comparative Fit Index (CFI) ≥ 0.95 (0.90–0.94 acceptable), a standardized root mean square residual (SRMR) ≤ 0.05 (0.05–0.10 acceptable), and a root mean square error of approximation (RMSEA) ≤ 0.05 (0.05–0.10 acceptable) [40].

#### Main analysis

All the following analyses were conducted with IBM SPSS Statistics 27.0. In cases were at least 80% of questions were answered, missing values were replaced by the mean value of the remaining items for that participant (*n* = 9). Categorical demographic variables (presence of anxiety, mood, behavioural disorders, presence of OCD, gender) were chi-square tested. For our continuous sociodemographic variable ‘age’, we conducted an analysis of variance.

**Fig. 1:**
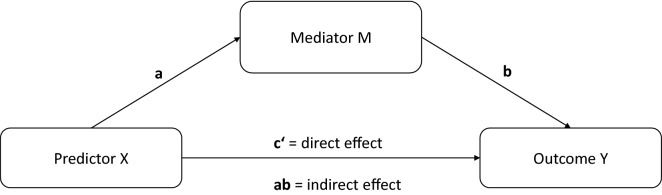
Exemplary mediation model

Since all our continuous main outcomes (coping score, use frequency for tobacco, alcohol, cannabis, MDMA, and stimulants) did not fulfil the criterion for normality (see Additional file 1: Table S1), non-parametric testing was applied. To predict the presence (yes/no) of past-year tobacco, alcohol, cannabis, MDMA, and stimulant use, five binary logistic regressions were calculated with group membership (noTEs, TEs, PTSD), and sociodemographic variables (other mental disorders, gender, age) that differed between the three groups as predictors, and the presence of past-year use of each substance as outcome. To control for differences in sociodemographic variables regarding the continuous outcomes (coping score, use frequency for tobacco, alcohol, cannabis, MDMA, and stimulants) differences in these outcomes were calculated with the Mann–Whitney U test. If the Mann–Whitney U test was non-significant, group (noTEs, TEs, PTSD) differences in substance use frequency were calculated with a Kruskal–Wallis test. In case the Mann–Whitney U test detected significant differences for sociodemographic variables, Quade’s test [41] was used to perform a non-parametric test while controlling for a covariate. Additionally Spearman’s correlation coefficient *ρ* was calculated for the association between coping score and use frequency for each substance for which significant group differences could be detected. Mediation analyses for all substances for which significant group differences were detected, were performed with the PROCESS macro [42]. PROCESS provides both a significance test and an effect size estimate with 95% confidence interval for the mediational effect of mediator variable M (‘coping score’) on the relationship between a predictor X (‘group membership’) and an outcome Y (‘substance use frequency’). This indirect effect (ab) describes by how much the relationship between X and Y (c’) is affected by the relationship between X and M (a), and the relationship between M and Y (b), see Fig. 1. The significance level for all analyses were set to α = 0.05.

## Results

### Sample description

Between-group differences in sociodemographic variables and the presence of mental disorders are shown in Table 1. Females were significantly underrepresented in the noTEs group [X^2^ (2) = 14.2, *p* < 0.001] while several co-occurring mental disorders were overrepresented in the PTSD group [X^2^ (2) = 14.0, *p* ≤ 0.007]. Based on group differences in the presence of OCD we excluded the *n* = 5 participants with an OCD diagnosis from the analysis, leaving *n* = 106 participants. Based on the sociodemographic differences we controlled for gender and presence of anxiety disorder in our main analysis.

**Table 1:** Demographic information about the three samples

	Total	NoTEs	TEs	PTSD	Group comparison	
					Test statistic	*p-*value
N (female)	111 (48)	31 (5)	42 (20)	38 (23)	*X*^*2*^*(*2) = 14.2	<0 .001^a^
Age (SD)	16.0 (1.3)	16.0 (1.3)	16.0 (1.2)	16.1 (1.3)	*F*(108) = 0.8	0.923
N with substance use disorders (%)					*X*^*2*^* (*3) = 2.9	0.816
Alcohol	45 (40.5%)	11 (35.5%)	18 (42.9%)	16 (42.1%)		
Cannabis	58 (52.3%)	15 (48.4%)	24 (57.1%)	19 (50.0%)		
MDMA	26 (23.4%)	4 (13.0%)	11 (26.2%)	11 (29.0%)		
Stimulants	25 (23.4%)	3 (9.7%)	12 (28.6%)	8 (21.1%)		
N with psychotic disorders (%)	4 (3%)	0	1 (2%)	3 (8%)	*X*^*2*^* (*2) = 3.4	0.187
N with anxiety disorders (%)	20 (18%)	2 (6%)	4 (10%)	14 (37%)	*X*^*2*^* (*2) = 14.0	< .001^a^
N with mood disorders (%)	62 (56%)	15 (48%)	20 (48%)	27 (71%)	*X*^*2*^* (*2) = 5.4	0.067
N with behavioural disorders (%)	57 (51%)	13 (42%)	19 (45%)	11 (29%)	*X*^*2*^* (*2) = 2.2	0.341
N with obsessive–compulsive disorder (%)	5 (5%)	0	0	5 (13%)	*X*^*2*^* (*2) = 10.1	0.007^a^

*OCD* Obsessive–Compulsive disorder; *MDMA* 3, 4 Methylenedioxymethamphetamine

^a^Significant at the 0.05 level; *anxiety disorders*; general anxiety disorder, panic disorder, agoraphobia, separation anxiety disorder, social phobia, specific phobia; *mood disorders*, major depression and bipolar disorder; *behavioural disorders*; attention deficit hyperactivity disorder, conduct disorder, oppositional defiant disorder

### Confirmation of the use motive questionniare

Data from the use motive questionnaire was suitable for factor analyses with Kaiser–Meyer–Olkin coefficient = 0.83, a significant Bartlett test with *p* < 0.001 and a measures of sample adequacy coefficient of 0.80. In the exploratory factor analysis, items had satisfying communalities after extraction. The only exception was item 18 (‘I am scared to loose friends when I stop using drugs.’) from the’social motives’ scale (*h*^2^ = 0.041) that was therefore not used within the following CFA analysis. Solutions with either two factors (indicated by Parallel Analysis and MAP test) or three factors (indicated by Kaiser-Guttman criterion) were suggested. The two-factor model explaining 63% of variance and producing one cross-loading item was deemed improper for further analysis as it collapsed all but the ‘social’ items into one large factor. The ‘empirical’ three-factorial model was selected for further CFA testing given that it was more theoretically sound by reproducing most of the theorized item assignments and that it explained variance to a rather large degree (67%). However, it deviated from the ‘theoretical’ three-factor model by having two ‘coping’ items with cross-loadings on the third factor, and one ‘other’ item loading on ‘coping’ only. A ‘combined’ model was defined for further CFA analysis based on the ‘empirical’ model; however, the ‘other’ item was therein assigned to the ‘other’ scale in order to have all scales more theoretically sound.

In the CFA*,* the ‘combined’ model was deemed appropriate within our sample due to mostly acceptable model fit values, with Χ^2^/df-ratio = 2.05, CFI = 0.94, SRMR = 0.05, but RMSEA 90%CI = 0.08-0.18, see Additional file 1: Table S2. Fit indices for the theoretical and empirical models were in comparable ranges, but less advantageous. Therefore, we assume that all four items theorized to measure a common construct (presumably ‘coping’) indeed measure a common construct that differed from what other motive items measure. However, two of those items covering substance use due to stressful events, or substance use due to inner tension, were also cross-loading on another factor.

### Substance use

The logistic regression models showed that the presence of past-year MDMA use (*b* = 0.66, *p* = 0.034, *OR* = 1.94) was significantly predicted by group membership (noTEs, TEs, PTSD) when controlling for sex and presence of anxiety disorders, while there were no relationships between group membership and the presence of tobacco use (*b* =—0.19, *p* = 0.721, *OR* = 0.83) alcohol use (*b* = 0.40, *p* = 0.289, *OR* = 1.49), cannabis use (*b* = − 0.38, *p* = 0.392, *OR* = 0.68), or stimulant use (*b* = 0.42, *p* = 0.196, *OR* = 1.52).

The presence of anxiety disorders was not associated with the frequency of past-year use of tobacco (*U* = 685, *p* = 0.746, η^2^ = 0.001), alcohol (*U* = 402, *p* = 0.687, η^2^ = 0.002), cannabis (*U* = 395, *p* = 0.193, η^2^ = 0.018), MDMA (*U* = 407, *p* = 0.080, η^2^ = 0.026), or stimulants (*U* = 225, *p* = 0.240, η^2^ = 0.015). Similarly, both genders did not differ in past-year tobacco use frequency (*U* = 1005, *p* = 0.161, η^2^ = 0.011), alcohol use frequency (*U* = 867.5, *p* = 0.351, η^2^ = 0.010), cannabis use frequency (*U* = 967, *p* = 0.703, η^2^ = 0.002), MDMA use frequency (*U* = 897, *p* = 0.197, η^2^ = 0.014), or stimulant use frequency (*U* = 609, *p* = 0.561, η^2^ = 0.004).

The past-year frequency of MDMA use differed between the noTEs, TEs and PTSD group (*H* (2) = 7.2, *p* = 0.027, η^2^ = 0.058), but no differences were detected regarding the past-year frequency of tobacco (*H* (2) = 1.6, *p* = 0.457, η^2^ = 0.004), alcohol (*H* (2) = 2.8, *p* = 0.256, η^2^ = 0.008), cannabis (*H* (2) = 4.9, *p* = 0.085, η^2^ = 0.033), or stimulant (*H* (2) = 1.3, *p* = 0.512, η^2^ = 0.009) use. Details regarding the patterns of substance use in the different groups are displayed in Table 2.

**Table 2:** Group differences in substance use and coping

	Total (*n* = 106)	NoTEs (*n* = 31)	TEs (*n* = 42)	PTSD (*n* = 33)	Group comparisons		
					Test statistic (SE)	*p*-value	Effect size
Mean coping score (SD)	5.4 (5.6)	3.2 (4.0)	5.1 (5.3)	7.6 (5.6)	*F* (103) = 5.77	0.004^a^	η^2^ = .101
Number of participants having used the substance in the past year							
Tobacco (*n* = 14 missings)	89 (83%)	25 (80%)	36 (86%)	29 (88%)	*b* =—0.19 (0.38)	0.721	*OR* = 0.83
Alcohol (*n* = 13 missings)	75 (81%)	20 (69%)	32 (89%)	23 (82%)	*b* = 0.40 (0.38)	0.289	*OR* = 1.49
Cannabis (*n* = 12 missings)	82 (87%)	26 (90%)	32 (86%)	24 (86%)	*b* = -0.38 (0.45)	0.392	*OR* = 0.68
MDMA (*n* = 12 missings)	41 (44%)	07 (23%)	18 (49%)	16 (59%)	*b* = 0.66 (0.31)	0.034^a^	*OR* = 1.94
Stimulants (*n* = 11 missings)	32 (34%)	05 (17%)	17 (44%)	10 (37%)	*b* = 0.42 (0.32)	0.196	*OR* = 1.52
Number of days of substance use per month over the past year (SD)							
Tobacco (*n* = 14 missings)	25.0 (10.1)	23.0 (11.7)	26.2 (8.4)	25.3 (10.1)	*H* (2) = 1.6	0.457	η^2^ = .004
Alcohol (*n* = 11 missings)	7.6 (10.1)	6.0 (9.3)	7.4 (10.6)	9.6 (10.4)	*H* (2) = 2.8	0.256	η^2^ = .008
Cannabis (*n* = 10 missings)	15.7 (12.1)	17.1 (12.9)	17.7 (11.8)	12.4 (11.5)	*H* (2) = 4.9	0.085	η^2^ = .033
MDMA (*n* = 9 missings)	2.3 (5.2)	0.9 (2.5)	1.6 (2.5)	4.4 (8.1)	*H* (2) = 7.2	0.027^a^	η^2^ = .058
Stimulants (*n* = 28 missings)	3.7 (8.0)	4.6 (9.0)	2.3 (6.1)	4.3 (9.0)	*H* (2) = 1.3	0.512	η^2^ = .009

*MDMA* Methylenedioxymethamphetamine, *SD* Standard deviation; *OR* Odds ratio

^a^Significant at the .05 level

### Coping score

While the presence of anxiety disorders was not associated with differences in coping score (U = 613.5, *p* = 0.340, η^2^ = 0.008), the two sexes showed a significant difference in coping score (U = 993, *p* = 0.012, η^2^ = 0.057); making it necessary to control for this variable in calculating the association between group membership (noTEs, TEs, PTSD) and coping score. While controlling for sex, the three groups differed significantly in terms of coping scores (*F* (103) = 5.77, *p* = 0.004, η^2^ = 0.101), with level of reported coping motive being highest in the PTSD group and lowest in the noTEs group. Additionally, the frequency of past-year MDMA use correlated significantly and positively with coping score (*ρ* = 0.287, *p* = 0.004).

### Meditation analysis

**Fig. 2:**
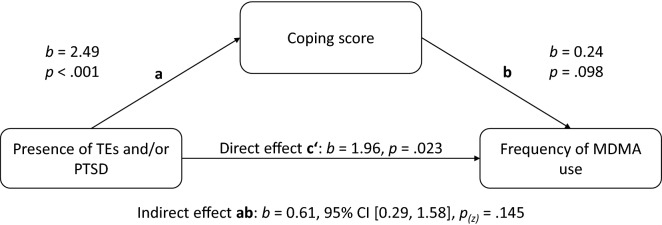
Mediation model for the effect of PTSD group on MDMA use frequency mediated by coping score

The mediation analyses for the effect of group membership (noTEs, TEs, PTSD) on past-year MDMA use frequency resulted in an indirect effect of coping score (*b* = 0.61, 95% CI [0.29, 1.58], *p* = 0.145), see Fig. 2. Although the *p-*value is larger than the α-level of 0.05, the CI not including zero indicates a true effect. That is, coping motives mediate how the presence of TEs and/or PTSD is associated with the past-year frequency of MDMA use in adolescents treated for SUD.

## Discussion

In the present study, we investigated the relationship between lifetime TEs and current PTSD diagnosis, substance use frequency and coping motives related to substance use in German adolescent SUD patients. We found that adolescents with co-occurrence of SUD and PTSD reported stronger coping motives and, in turn, a higher frequency and likelihood of MDMA use in the past year. Associations were specific to MDMA; they did not exist for tobacco, alcohol, cannabis, or stimulants.

Similarly to previous research from our group investigating past-month substance use [25], a co-occurrence of PTSD and SUD was associated with a higher frequency of past-year MDMA use in adolescents seeking treatment for SUD. Previous research supports our finding in so far as MDMA use has been associated with the presence of general psychopathological symptoms [43], use of multiple psychoactive substances [44] and with higher rates of PTSD [45]. However, in studies with adults, PTSD has mostly been associated with the use of alcohol [5, 46, 47] when compared to non-using populations. Since our sample showed high levels of alcohol use (amongst other substance use) as well, it might be more accurate to say that we showed MDMA use to be associated with PTSD in a sample of adolescents with high levels of co-occurring substance use.

A first possible explanation for this relationship between MDMA use and PTSD might be a detrimental effect of MDMA use on PTSD development. Specifically, a more frequent MDMA use might lead to an escalation of sub-clinical PTSD symptoms until the criteria for a PTSD diagnosis are fulfilled. Support for this line of argument could be found in previous research that has shown that psychopathological symptoms like depression or aggression might develop after MDMA use [43, 45]. Another explanation, supported by the results of our mediation analysis, suggests that an increased use of MDMA is observed in patients with PTSD symptoms because of a stronger need to cope with PTSD-related symptoms. Our analysis has shown that part of the effect of TEs/PTSD on MDMA use frequency is explained by the level of reported coping motives for substance use. Plainly, if a higher frequency of coping motives was reported by a patient, the effect of PTSD on MDMA use frequency was increased as well. This finding is in line with the hypothesis that substance use might serve as a coping measure [22, 23]. This specific relationship of PTSD symptoms with MDMA has been shown previously [48–50], while MDMA use in recreational non-pathological users is mainly related to enhancement or expansion motives instead of coping [51]. Specifically, Jansen [48] described a case report of a patient with PTSD who unambiguously ascribes his symptom relief to the acute effects of MDMA. Further, Scott et al. [50] have shown that higher levels of coping motives are related to higher levels of MDMA use which is in line with our correlational analysis as well. Additionally, their research and one other study support our conclusion, that it are PTSD symptoms specifically that are related to increased MDMA use [31], not TEs in general [30, 50]. Furthermore, Moonzwe et al. [49] showed in great detail how, for young adults, MDMA use has been described by users as particularly effective in terms of coping with negative consequences of TEs. However, the authors also point out that this relationship is present only in participants who did not receive satisfactory mental health treatment, while in well-treated participants, MDMA use was not related to a significant coping effect [49]. This literature and our results may be relevant regarding recent systematic reviews [52–54] and phase-3 studies [55] indicating that MDMA-supported psychotherapy might be beneficial in PTSD patients. While the effects of MDMA-supported psychotherapy seem promising in adult patients with treatment-resistant PTSD, often military veterans [56], there are no studies so far investigating this process in adolescent patients. Importantly, our participants use MDMA in recreational settings (clubs, festivals, raves, etc.), without psychotherapeutic support and mostly in form of pills with little to no knowledge of its contents, indicating a large difference to therapeutically administered MDMA.

It remains speculative why specifically MDMA was involved in coping activities as compared to other substances with anxiolytic and sedative effects such as alcohol (which is known from adult studies) or opiates with their potentially more symptom-relieving properties [57]. One issue is that alcohol is more available and more commonly used among adolescents in the study region compared to MDMA [58]. Its use might simply be much too high and prevalent in our sample, resulting in a ceiling effect that prevents us from detecting self-medicating patterns due to high use in non-self-medicating adolescents. Opiates, on the other hand, may not have been encountered by these patients, may have been less available compared to MDMA or more difficult to afford on a regular basis in the study region. In fact, only 3 of 201 adolescent patients in our institution reported any opiate use in the 12 months before admission [58].

While at first glance it might seem like MDMA-related coping is beneficial for these patients, this practice is also related to a variety of negative outcomes. Specifically, the relief from distress is thought to act as a negative reinforcement, increasing the likelihood for further use in the future and increasing the risk for the aetiology of a MDMA use disorder according to current learning theories related to SUDs [20, 21]. Likewise, reporting coping motives for substance use during adolescence is associated with higher rates of SUDs later in life [59], indicating that the patients we saw in our study, might go on to develop more severe patterns of substance use later on. Further, a coping motive is not equivalent to a successful symptom reduction. For example, some participants report that MDMA use is more of a temporary break from PTSD symptomatology instead of having any substantial effect beyond the acute high [49]. Finally, a reduction in substance is made much more difficult as long as a coping behaviour is in place, leading to higher rates of relapse in patients with this use pattern [31].

### Limitations

First, we did not use a validated measure to assess substance use motives. Our measure was based on a self-designed questionnaire that was available in our research group, which did assess use motives but was not specifically designed for this purpose. Consequently, our coping score might not reflect a measure of coping motives but instead might represent another unclear factor related to PTSD presence and MDMA use frequency. However, we did provide exploratory and confirmatory factor analyses for the questionnaire providing some preliminary support for it being psychometrically sound.

Second, our sample consisted entirely of treatment-seeking patients, which does not allow for generalizations of the relationship between MDMA use, coping motives, and TEs for MDMA substance users outside this clinical setting.

Third, our cross-sectional design does not allow for conclusions about causal relationships. While we argue that patients take MDMA to reduce PTSD symptoms and might therefore facilitate a development of a SUD, this is mere association. To determine a causal chain in this relationship longitudinal studies are needed.

Fourth, the three groups in our sample differed in terms of gender distribution and the presence of mental disorders. However, we controlled for these factors in our main analysis, and examined if they were associated with our main outcomes. Based on these analyses we concluded that the gender and psychopathological differences did not influence our main outcomes.

Fifth, our adolescent patients are mostly motivated externally (e.g. through parents) to participate in research, which resulted in a large number of questionnaires not being filled out.

Finally, our argument for a coping effect rests on MDMA relieving PTSD related symptoms. However, we did not ask participants to report coping motives specific for PTSD. Instead, participants reported general coping motives dealing with the relief of negative emotional states. Future research should take care to include measures that specifically ask if substances were used to reduce PTSD symptoms specifically.

## Conclusion

This study in German adolescent psychiatric patients showed that a co-occurring PTSD and SUD is related to higher MDMA use compared to patients without a co-occurring PTSD. This use was increased even when controlling for other substance use, gender, and comorbid disorders. Additionally, we showed that the effect of PTSD on MDMA use frequency is mediated by the level of coping motives, indicating that MDMA use might be higher in this population, partly because of a coping motive.

## Supplementary Information

**Additional file 1: Table S1.** Shapiro-Wilk test for normality of the 5 outcome variables. **Table S2.** Results from the confirmatory factor analysis. **Table S3.** The 10 use motive items from the self-designed questionnaire.

## Data Availability

The datasets used and analysed during the current study are available from the corresponding author on reasonable request.
