# Supplementary material for: Coping motives mediate the relationship between PTSD and MDMA use in adolescents with substance use disorders
Source: Addict Sci Clin Pract. 2022 Sep 4;17:46. doi: 10.1186/s13722-022-00329-y (PMC9441101; doi:10.1186/s13722-022-00329-y)
Supplement: Supplementary file 1 — Additional file 1: Table S1. Shapiro-Wilk test for normality of the 5 outcome variables. Table S2. Results from the confirmatory factor analysis. Table S3. The 10 use motive items from the self-designed questionnaire. [file 13722_2022_329_MOESM1_ESM.docx]

Coping motives mediate the relationship between PTSD and MDMA use in adolescents with substance use disorders

**Authors**: Lukas Andreas Basedow^1, 2^, Melina Felicitas Wiedmann^1^, Veit Roessner^1^, Yulia Golub^1^, Sören Kuitunen-Paul^1, 3^

**Affiliations:** ^1^TU Dresden, Dept. of Child and Adolescent Psychiatry, Faculty of Medicine, Dresden, Germany

^2^ Division of Clinical Psychology and Psychotherapy, Dept. of Psychology, Philipps-University of Marburg, Marburg, Germany

^3^ Chair for Clinical Psychology and Psychotherapy, Technische Universität Chemnitz, Chemnitz, Germany

**Corresponding Author**: L.A. Basedow, [lukas.basedow@ukdd.de](mailto:lukas.basedow@ukdd.de)

*Supplemental Table 1.* Shapiro-Wilk test for normality of the five outcome variables.

|  | Test statistic | *p*-value |
| --- | --- | --- |
| #Days of tobacco use | *W* (23) = .54 | <.001 |
| #Days of alcohol use | *W* (23) = .75 | <.001 |
| #Days of cannabis use | *W* (23) = .83 | .001 |
| #Days of MDMA use | *W* (23) = .64 | <.001 |
| #Days of stimulant use | *W* (23) = .55 | <.001 |
| Coping score | *W* (23) = .89 | .018 |
| Note: Days of substance use are the average number of days of substance use per month over the past year | | |

Supplemental Table 2. Results from the confirmatory factor analysis.

| **Model** | **Χ^2^(df)** | **Χ^2^/*df*-ratio** | ***p*-value** | ***CFI*** | ***SRMR*** | ***RMSEA* [90% CI]** |
| --- | --- | --- | --- | --- | --- | --- |
|  |  |  |  |  |  |  |
| **Theoretical model** | 64.09 (24) | 2.67 | <.001 | .91 | .09 | .16 [.11 – .21] |
| **Empirical model** | 45.79 (22) | 2.08 | .002 | .95 | .04 | .13 [.07 – .18] |
| **Combined model** | 49.33 (24) | 2.05 | .002 | .94 | .05 | .13 [.08 – .18] |
| *Notes*. Good [acceptable] model fit is indicated by Χ**^2^**/*df*-ratio < 2 [<3], *CFI* ≥ .95 [.90 - .94], *SRMR* ≤ .05 [.05 - .10], and *RMSEA* ≤ .05 [.05-.10 acceptable].  *CFI* = Comparative Fit Index. *RMSEA* 90% *CI* = 90% confidence interval of the root mean square error of approximation. *SRMR* = Standardized Root Mean Square Residual. | | | | | | |

Supplemental Table 3. The ten use motive items from the self-designed questionnaire [translated into English by LAB].

| **Original German item** | **English translation** | **Domain** |
| --- | --- | --- |
| Ich konsumiere Drogen, wenn ich in einer Stresssituation bin. | I use drugs when I am in a stressful situation. | Coping |
| Ich konsumiere Drogen, wenn ich angespannt bin. | I use drugs when I feel tense. |  |
| Ich konsumiere Drogen meistens wenn es mir nicht gut geht. | I mostly use drugs when I am not well. |  |
| Ich konsumiere Drogen meistens, damit es mir besser geht. | I use drugs to feel better. |  |
| Ich habe das Gefühl, wenn ich Drogen konsumiere bin ich cooler. | I feel like I am cooler when I use drugs | Social |
| Ich habe das Gefühl, wenn ich Drogen konsumiere, dann habe ich mehr Freunde. | I think I have more friends when I use drugs. |  |
| Ich habe Angst meine Freunde zu verlieren, wenn ich keine Drogen konsumiere. | I am scared to loose friends when I stop using drugs. |  |
| Ich fühle mich stärker unter dem Einfluss von Drogen. | I feel stronger under the influence of drugs. | Other |
| Ich fühle mich selbstbewusster unter dem Einfluss von Drogen. | I feel more confident under the influence of drugs. |  |
| Ich weiß nicht was ich sonst tun soll, als Drogen zu nehmen, wenn mir langweilig ist. | When I am bored, I do not know that to do except use drugs. |  |
